# The Human Dermis as a Target of Nanoparticles for Treating Skin Conditions

**DOI:** 10.3390/pharmaceutics15010010

**Published:** 2022-12-20

**Authors:** Javier Salazar, Thais Carmona, Flavia C. Zacconi, Diego Venegas-Yazigi, Claudio Cabello-Verrugio, Won Il Choi, Cristian Vilos

**Affiliations:** 1 Laboratory of Nanomedicine and Targeted Delivery, School of Medicine, Universidad de Talca, Talca 3460000, Chile; 2 Departamento de Química Orgánica, Facultad de Química y de Farmacia, Pontificia Universidad Católica de Chile, Av. Vicuña Mackenna 4860, Macul, Santiago 7820436, Chile; 3 Center for The Development of Nanoscience & Nanotechnology (CEDENNA), Universidad de Santiago de Chile, Santiago 8350709, Chile; 4 Departamento de Química Analítica, Química Física e Ingeniería Química and Instituto de Investigación Química “Andrés M. Del Rio” (IQAR), Universidad de Alcalá, 28805 Alcalá de Henares, Madrid, Spain; 5 Materials Chemistry Department, Faculty of Chemistry and Biology, University of Santiago of Chile (USACH), Santiago 9170022, Chile; 6 Institute for Biological and Medical Engineering, Schools of Engineering, Medicine and Biological Sciences, Pontificia Universidad Católica de Chile, Av. Vicuña Mackenna 4860, Macul, Santiago 7820436, Chile; 7 Center for Nanomedicine, Diagnostic & Drug Development (cND3), Universidad de Talca, Talca 3460000, Chile; 8 Laboratory of Muscle Pathology, Fragility and Aging, Faculty of Life Sciences, Universidad Andres Bello, Santiago 8370035, Chile; 9 Millennium Institute on Immunology and Immunotherapy, Faculty of Life Sciences, Universidad Andres Bello, Santiago 8370035, Chile; 10 Center for Bio-Healthcare Materials, Bio-Convergence Materials R&D Division, Korea Institute of Ceramic Engineering and Technology, 202, Osongsaengmyeong 1-ro, Osong-eup, Heungdeok-gu, Cheongju 28160, Chungbuk, Republic of Korea

**Keywords:** skin, dermis, nanoparticles, drug delivery, metal-organic frameworks (MOFs)

## Abstract

Skin has a preventive role against any damage raised by harmful microorganisms and physical and chemical assaults from the external environment that could affect the body’s internal organs. Dermis represents the main section of the skin, and its contribution to skin physiology is critical due to its diverse cellularity, vasculature, and release of molecular mediators involved in the extracellular matrix maintenance and modulation of the immune response. Skin structure and complexity limit the transport of substances, promoting the study of different types of nanoparticles that penetrate the skin layers under different mechanisms intended for skin illness treatments and dermo-cosmetic applications. In this work, we present a detailed morphological description of the dermis in terms of its structures and resident cells. Furthermore, we analyze the role of the dermis in regulating skin homeostasis and its alterations in pathophysiological conditions, highlighting its potential as a therapeutic target. Additionally, we describe the use of nanoparticles for skin illness treatments focused on dermis release and promote the use of metal-organic frameworks (MOFs) as an integrative strategy for skin treatments.

## 1. Skin Function and Composition 

The skin is the human body’s largest organ, and its primary function is to prevent any damage raised by harmful microbes, UV radiation, weather, pollution, or other assaults from the external environment that could affect the body’s internal organs [1]. The skin also has a social role because its appearance can determine how people feel towards or judge each other socio-economically, as sexual partners, or even to get a job [2]. Skin affections represent the fourth of global, not lethal, disease burdens [3], and skin-related diseases have prevalence and cost equivalent to other significant public health concerns, such as cardiovascular disease and diabetes [4]. 

**Figure 1:**
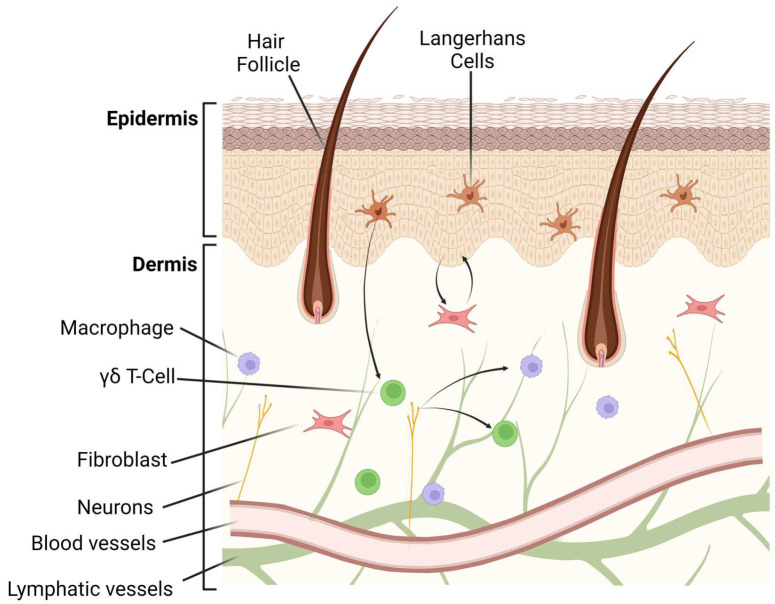
Illustration of skin components and their cell distribution.

The histological analyses of the skin show the dermis as the more extensive section localized between an outer layer named the epidermis and a deep section called the hypodermis. The dermis is composed mainly of fibrous tissue, such as collagen, elastic fibers, and glycosaminoglycans that, together with diverse structures such as the nerve terminals, glands, blood vessels, and follicles, provide sensorial and protective properties to the skin, as shown Figure 1 [5,6].

The skin is exposed daily to countless factors that undermine its protective and structural properties as a peripheral organ. Those unique factors for each individual, such as metabolism, genetics, and epigenetics, can be classified as intrinsic factors; in contrast, those standard elements that affect the group of individuals like environmental conditions (UV exposure, pollution, or weather) or lifestyle (nutrition, smoking, stress or lack of sleep) can be categorized as extrinsic factors [7]. Both intrinsic and extrinsic factors and aging impair keratinocyte and fibroblast’s ability to maintain the skin’s homeostasis, and some complications, such as inflammation-related illness, autoimmune pathologies, structural disorders, and cancer, can arise [8].

## 2. Dermis

The dermis represents the main section of the skin. The dermis can be classified into papillary and reticular dermis based on the components and morphology of each section [9]. The papillary dermis is the thinner area of the dermis and exhibits an intertwining with the epidermis [5]. Compared to the reticular dermis, the papillary dermis presents a distribution of less compact fibrous components, which allow the presence of blood vessels that nourish the epidermis [10,11]. Furthermore, the Schwann cells on the papillary dermis project their dendrite to the epidermis, making sense through the skin [12]. Conversely, the compact distribution of fibrous content on the reticular dermis gives the stretching and resilience resistant properties to resist the deformation forces applied to the skin [13]. The contribution of the dermis to skin physiology is significant; therefore, deep knowledge about the composition and distribution of their components is critical, as well as the interaction of the cellular population and how they are affected under diverse pathologies that affect the skin. In this sense, it is critical to evaluate the role and relation between each key component of the dermis to generate novel nanotechnology-based systems for treating skin conditions.

## 3. The Cellular Population of the Dermis

Those cellular types on the dermis hold crosstalk that coordinate protection against injury, physical stimuli, or pathogen assault. The cellular population of the dermis also coordinates skin appendage formation, such as hair follicles, sebaceous glands, and sweat glands, that contribute to the protection and thermoregulation of the body. Furthermore, the specialized cells on the dermis as the nervous terminals sense temperature, pain, and mechanics’ force. The crosstalk between the different cell types depends on the distribution and maintenance of the fibrillar scaffold in the dermis. The fibroblast is mainly responsible for their synthesis and restructuration [14].

### 3.1. Fibroblast

Fibroblasts are mesenchymal cells with different origins, locations, and functions that synthesize and maintain the extracellular components [15]. The dermal fibroblasts can be divided into subtypes, such as papillary fibroblast, reticular fibroblast, dermal papilla (DP), and dermal white adipose tissue (DWAT) [15,16,17]. The cellular markers that allow for classifying the fibroblast subpopulations fluctuate through embryo development. However, some features remain specific to papillary, reticular, or DWAT. Studies on homologous models such as mice demonstrate that fibroblast on the skin can be isolated through the surface marker FAP and CD90 [18,19]. 

Interestingly, different from the mouse model, the human skin presents a fibroblast population gradient where FAP and CD90 expression change depending on the dermis section. In the human skin, the papillary fibroblast can be identified as FAP+ CD90−, the reticular fibroblast as FAP+ CD90+, and FAP− CD90+ pre-adipose reticular fibroblast [20]. The difference in these fibroblast populations is not exclusively on cellular markers that describe their anatomical locations. The fibroblast subpopulation presents a different gene and protein expression that determines how the fibroblast responds to environmental stimuli [21]. Under specific signals such as adipogenesis-induced medium reticular fibroblast, FAP− CD90+ can differentiate to adipocyte, whereas papillary fibroblast FAP+ CD90− cannot be differentiated. Sequencing studies also show that papillary fibroblast has enhanced collagen type VII and type III expressions related to papillary dermis formation, blood vessels, and dermo-epidermal junction (DEJ). In contrast, reticular fibroblast presents an enhanced expression of extracellular matrix (ECM)-related genes such as LOXL3, a lysyl oxidase related to elastin and collagen synthesis [21]. The difficult task of studying the fibroblast subpopulation on dermal skin arises because fibroblast changes their surface markers and protein expression profile under cell culture conditions [22].

When the skin’s integrity is compromised, as in a wound, the fibroblast and immune cells are recruited by specific cytokines TGFβ-1, IL-1, IL-6, and chemokines secreted by damaged keratinocyte and platelets [23] to produce ECM components and guide the healing process. In some cases, the fibroblast response is overregulated, leading to an aberrant scarring process such as hypertrophic scarring or keloid [23]. The difference between both fibrotic processes is that keloid expands beyond the limits of the original wound, and the amount of fibrillar content increases over time [24]. Nevertheless, both fibrotic processes present an increased presence of activated fibroblast, denominated myofibroblast, that remodel and secrete an increased amount of collagen type I [25]. Furthermore, the conversion to myofibroblast could be mechanical regulation related to collagen fiber contraction [26] and the presence of cytokines TGFβ-1 and IL-11 secreted by papillary fibroblast CD39+ [27]. A recent study demonstrates that the conversion to myofibroblast by TGFβ-1 signaling is mediated by the expression of the Engrailed 1 (EN1) transcription factor [28]. Moreover, the indirect inhibition of EN1 through the inhibition of yes-associated protein (YAP) by verteporfin prevents fibroblast activation and conduces to a scarless wound repair [29].

Together with the wound repair process, the fibroblast function is related to the skin’s homeostasis. Skin homeostasis is affected by aging, a complex multifactorial process involving all human beings. Skin aging is affected by extrinsic and intrinsic factors and is characterized by a loss of skin functions such as impaired barrier function, loss of stiffness, the impaired healing process, and altered immunological response [7]. On the fibroblast, the aging process is characterized by the increased production of reactive oxygen species (ROS) generated by ultraviolet radiation overexposure through life [30]. The increased ROS content promotes mutations related to desoxyribonucleic acid (DNA) damage on the fibroblast [31]. Furthermore, the aged fibroblast produces IL-6 and IL-8 cytokines, which are involved in chronic inflammation [32]. Moreover, the aged fibroblast increased the secretion of matrix metalloproteinase-1 (MMP-1), which degrades the collagen content of the dermis, decreasing the stiffness of the skin [33]. The loss of rigidity on the dermis alters the proliferation of keratinocytes on the epidermis [34] and reinforces the aged phenotype on fibroblast, promoting the secretion of MMP-1 [35,36]. These antecedents demonstrate that the fibroblast is present as a critical cell in the dermis physiology, and their misfunction could enhance pathological processes such as fibrosis, keloid, impaired barrier function, and dermatoporosis [37]. 

### 3.2. Immune Cells

The primordial function of the skin is a barrier achieved through the stratum corneum (SC) of the epidermis and the immune cells on all skin. The heterogeneous presence of immune cells on the skin has been reviewed [38]. However, the dermis remains in other heterogenic populations of immune cells that complement epidermal immune cells’ function. The population present on the dermis have linfoid or myeloid origin; some are mast cells, dendritic cells (DC), T CD4+, T CD8+, γδ T cell, and memory-resident T cells (Trm) [6].

The interaction of sphingosine-1-phosphate (S1P) and the sphingosine-1-phosphate receptor 1 (S1PR1) present on the membrane of immune cells are the primary signal for the migration of resident immune cells to the lymphatic nodule (LN) present on skin [39]. Otherwise, the interaction of S1PR2 and CD 69 is responsible for the lymphatic γδ T cell resident on the dermis. The interactions of S1PR2 with its ligand inhibit the migratory signal given by S1PR1, whereas CD 69 down-regulates the expression of S1PR1 on the lymphocyte membrane [40]. That mechanism suggests a close regulation between the cells that reside on the dermis and those populations of cells that migrate on the lymphatic nodule. Under this mechanism, diverse lymphocytes, such as Trm CD 8+, patrol the dermis and the epidermis regularly [41]. Trm CD8+ have a dendritic-like shape that lets them search for antigens between the tight interaction of keratinocytes on the epidermis. Upon recognizing an antigen, Trm CD8+ shift their form to a spheric-like. Additionally, they began the synthesis and secretion of interferon γ (INF-γ) that mediate the recruiting of other immune cells, starting a local immune response [42]. Another lymphocyte that mediates the inflammatory response against environmental allergens is γδT cells, which are present on the dermis from three days after born and could be residents of Dermis (Vγ 5+ subtype) or surveillance the skin-surrounded environment (Vγ 4+ subtype) such as skin LN. The IL-17A secreted by γδ T cells are essential in the inflammatory response, and their dysregulated secretion is a common factor in inflammatory diseases, such as psoriasis, atopic dermatitis, and contact allergies [43].

Dendritic cells (DC) and mast cells are myeloid-derived immune cells that are found to be closely related to neuronal terminals and blood vessels of the dermis [44]. Nerve terminals secrete neuropeptide that stimulates the secretion of IL-23 from DC, and its secretion stimulates the production of IL-17A on γδ T cells, which mediates a neuronal immune response against viruses such as herpes simplex [45]. However, mast cells form a physical synapse with unmyelinated C-fibers, and they have bidirectional communication with neurons in the skin [46]. Mast cells-derived TNF and nerve growth factor (NGF) stimulate neuronal elongation on the dermis. The crosstalk between mast cells and DC mediates the transition between innate and adaptative immune responses. DC can recognize nickel (Ni) with solid affinity and then migrate to the closed LN for beginning the antigen presentation, and it has been speculated that this interaction is responsible for the allergic response to Ni [47].

In some cases, the skin’s immune response can be altered and lead to disorders like omenn syndrome, vitiligo, psoriasis, atopic dermatitis, or allergic contact dermatitis. In immune deficiency syndrome as omen syndrome, where a lack of T or B cells occur, a penetration into the Dermis by Langerhans cells exists. This immune deregulation generates some skin conditions such as erythema and alopecia; the last one is caused by an immune attack over the hair follicle on skin [48,49,50]. 

## 4. Dermis as a Target for Nanotechnology-Based Treatments 

Nanotechnology intended for dermatology is a domain of research in constant progress. However, less than 1% of the nanoparticles under clinical trials focus on skin conditions, which include skin illness treatment, dermo-cosmetic, and wound care devices [51,52]. Moreover, transdermal applications for vaccination or systemic pathology treatment have been developed due to the vascular distribution of the dermis [53,54]. Those initiatives bypass the hepatic passage of drugs through oral administration or avoid utilizing needles during vaccination, which improves patients’ adherence to the different treatments. Indeed, the dermis represents a robust administration route to develop nanoparticles for medical applications, improving immunization through the activations of the dendritic cells and as a pathway to deliver drugs or nutrients to improve systemic or localized diseases.

### 4.1. Skin Penetration of Nanoparticles

**Figure 2:**
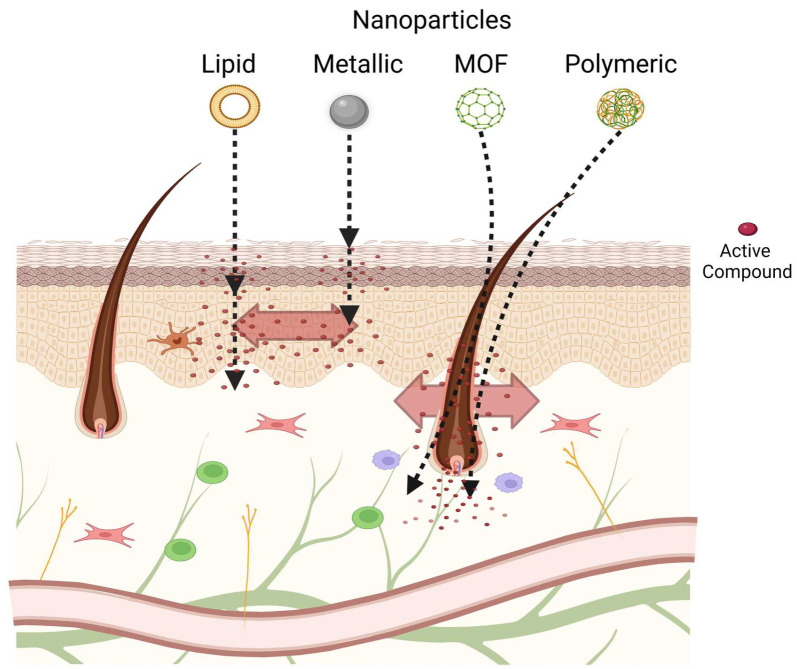
Scheme of potential skin penetration routes of different types of nanoparticles.

Regardless of the nanoparticle employed, the first obstacle is to penetrate across the skin layers, particularly the stratum corneum. The nanoparticles can penetrate the skin by following one of these routes: (1) The intracellular (transcellular) route, (2) the intercellular route, or (3) the appendage route, as displayed in Figure 2 [55]. On intact skin, the transcellular and intercellular routes can be reached using permeation enhancers that disrupt the integrity of the stratum corneum [56]. An example of the effect of an enhancer was illustrated in a study that analyzed the impact of the surface charge of nanoparticles on skin penetration prepared using different solvents. Despite the surface charge, the particles formulated in water were staked on the SC, whereas the particles prepared with ethanol/water crossed the SC and reached the dermis. The effect on the penetration of nanoparticles was not enhanced when ethanol was applied before the nanoparticles [57]. These researchers hypothesized that the ethanol drags the nanoparticles across the SC, reaching the viable epidermis where the nanoparticle can defund depending on their surface charge. The penetration of particles in the skin increases when skin integrity is compromised, such as through photodamage, atopic dermatitis, psoriasis, and dryness. Skin damaged by UVA radiation presents an increased skin permeation of nanoparticles [58,59].

The skin appendages are the sweat gland and the pilosebaceous unit, including the sebaceous gland and hair follicle [60]. The hair follicles act like ducts that connect the outer environment with the dermis. The dermal papilla in the deepest section of the hair follicle is associated with capillary loops and lymphatic vessels [61,62,63]. The high content of vessels associated with the dermal papilla transforms the trans appendage route into a critical target to reach systemic drug delivery or immunization through the skin. Nanoparticle penetration through hair follicles depends on the size of nanoparticles, the medium’s viscosity, and the hair’s movement [64,65]. The theoretical model denominated Ratchet effect explains the influence of these physicochemical properties. Based on the theoretical model, the ideal size of nanoparticles to reach the deepest part of the hair follicle is 600 nm. These theoretical sizes concord with the results obtained by other research where the nanoparticles with size between 400–600 nm present the deepest penetration on hair follicles [66,67].

### 4.2. Effect of Nanoparticles over the Dermis

The literature reports numerous works describing nanoparticle formulation intended for topical applications [51,68,69,70]. Compared to topical treatments, transdermal represents an effective transport from the intact skin into the systemic circulation to treat various chronic diseases. Transdermal treatments offer better patient compliance than more invasive alternative routes avoiding liver metabolic. However, only a limited number of drugs are enough small and lipophilic to pass the skin barrier [71]. Nanocarriers made of lipids, metals, or polymers have been developed to increase the penetration of drugs or vaccines and control drug release by targeting specific areas of the skin [72]. Numerous nanoparticles have been developed, some of which are covered in Table 1. 

#### 4.2.1. Inorganic Nanoparticles

Inorganic nanoparticles in dermatology comprise metal/metal oxide particles, carbon nanotubes, silica-based nanoparticles, and quantum dots, among others. Those types of nanoparticles are focused on treating cutaneous wounds, particularly in preventing and treating bacterial and fungal infections, and against the harmful effects of the sun as UV blockers. Additionally, they present chemical and thermal stability in the delivery systems; moreover, it can be exploited for simultaneous imaging and treatment. Tak et al. demonstrated shape-dependent skin penetration of Ag-NPs through different layers of the skin, indicating skin penetration of AgNPs through intercellular pathways [82]. Inorganic nanoparticles can be used alone, or in combination with polymers as composites [83] Muchova et al. aimed to provide an antibacterial effect using selenium nanoparticle (SeNP) adsorbed into a scaffold composed of chitosan, collagen, and thermostable fibroblast growth factor 2 (FGF2-STAB^®^). Those scaffold applications demonstrated a controlled release of SeNP in the dermis, lowering the ROS level and promoting wound regeneration [84]. Over the years, the application of gold nanoparticles as a drug carrier for skin drug delivery has attracted increasing attention because of their unique properties and versatility [85]. For example, Niu et al. synthesized gold nanoparticles conjugated with a peptide and cationic polymer (polyethelyimine, PEI) conjugated (AuPT) that could interact with a pDNAs encoding the miRNA-221 inhibitor gene into cationic nanocomplexes and penetrate through the intact stratum corneum [86]. Some of the inherent properties of metals or metallic oxides, such as magnetism, can be exploited. Yue-feng Rao et al. demonstrated that epirubicin covalently modified SPIONs (superparamagnetic iron-oxide nanoparticles) could be used as transdermal vectors as they could circumvent the stratum corneum via follicular pathways and reach the reticular dermis [76]. Ramadan et al. presented a different approach [87]. They used a photothermal ablation-enhanced transdermal drug delivery methodology on hollow copper sulfide nanoparticles (HCuSNPs). This technique induces skin perforations by a modulated laser that can induce localized thermal ablation of the SC, facilitating the penetration of the particles to the deeper skin layers. This skin disruption by HCuSNP-mediated photothermal ablation significantly increases the permeability of human growth hormone. Carbon nanodots are emerging as potential delivery systems because of their water solubility, chemical inertness, low toxicity, ease of functionalization, and resistance to photobleaching [88]. Bankoti and col. used carbon nanodots adsorbed into decellularized dermis to reduce the local ROS and to promote cellular recruitment and regeneration of wounds [89].

#### 4.2.2. Polymeric-Based Nanoparticles

Polymeric nanoparticles are one of the most attractive topics of research to be used as potential topical transdermal nanocarriers. Polymers have the advantages of low toxicity, biocompatibility, and biodegradability. Popular polymer materials used for delivery purposes are collagen, chitosan, poly(-lactic-co-glycolic) acid (PLGA), polycaprolactone (PCL), and dextran [90].

Chitosan-coated PLGA and bare PLGA with similar-sized but opposite surface charges have been studied by Mittal et al. [91]. They used ovoalbumin (OVA) to evaluate delivery efficacy. They found two–three times higher follicular penetration of NPs than pure OVA solution. These results paved the way for using polymer nanoparticles in formulations used in vaccines. They emphasized the potential of the trans follicular route to the administration of the drug to reach most internal layers of the skin. The application of nanoparticles synthesized with human recombinant keratin has promoted collagen synthesis and angiogenesis and improved wound healing mechanisms [92]. Other researchers have used a multifactorial approach using a polysaccharide-based hydrogel containing exosomes charged with interfering microRNA (miRNA); that complex formulation effectively relieves wound skin from UV damage, promoting the angiogenesis and regeneration of the skin appendages [93]. When the skin’s barrier function is compromised, different pathogens such as *Candida* spp., *Pseudomonas* spp., or *Staphylococcus* spp. can promote a permanent infection when they reach the dermis. An alternative to face this infection generated by candida spp. was to improve the biodistribution of the antimycotic itraconazole using absorbable microneedles charged with nanocrystal [94]. Furthermore, nanocrystal of azithromycin has been developed to treat Lyme disease generated by tick bite infection [95]. Recently, Kim et al. studied pH- and temperature-sensitive double cross-linked hydrogels consisting of poly(N-Isopropylacrylamide (PNIPAM) and Hyaluronic Acid (HA) as a transdermal drug delivery carrier of luteolin for its applicability to alleviate psoriasis. The in vitro skin permeation experiments showed that hydrogel effectively delivers luteolin to the epidermis and dermis. Jeong et al. evaluated the applicability of Carboxymethyl Chitosan/2-Hydroxyethyl Acrylate (CmCHT-g-pHEA) hydrogels as a transdermal delivery system. In addition, they confirmed that the CmCHT-g-pHEA hydrogels temporarily interferes with skin barrier function through skin hydration improving the skin penetration to lower layers of skin of nobiletin loaded into the hydrogel matrix [96]. Other researchers, using a multifactorial approach based on polysaccharide-based hydrogel containing exosomes charged with interfering microRNA (miRNA), demonstrated an effectively relieves of wound skin caused by UV damage, thus promoting angiogenesis and improving the regeneration of the skin appendages. Moreover, silver nanoparticles have been used to improve the performance of wound healing matrix composed of biopolymers such as polyvinyl alcohol (PVA), collagen, and hyaluronic acid [97]. From another perspective, towards the control of the fibrosis process, the use of nanoparticles of PLGA charged with pioglitazone decreases the TGF-β signal produced during skin fibrosis in scleroderma patients [98]. The immune cells in the dermis are essential in developing inflammatory diseases. It has been reported that a nanogel composed of hyaluronic acid and β-glucan efficiently activates the dendritic cells when incorporating an immunomodulator such as ovalbumin (OVA) [99]. Additionally, silibinin, a flavonoid that presents antioxidant and anti-inflammatory actions, has been delivered into the dermis using nanocapsules supported in a polymeric matrix to treat dermatitis [79]. Furthermore, nanoparticles of silica functionalized with PDMA (poly(2-(dimethylamine) ethyl methacrylate) were used to scavenge the cellular free DNA present in psoriasis, improving the symptoms in a murine model [100]. In addition, the neuronal component in the dermis has been targeted to improve the treatment of peripheral neuropathic pain using nanoparticles loaded with capsaicinoid and supported into hydrogel based on chitosan. This system’s probe has increased permeability and a higher biodistribution of capsaicinoids into the dermis [101]. Another application of the polymeric system was researched by Sanad et al., who prepared a chitosan-HA/Andrographolide nanocomposite scaffold. When applied to second-degree burn wounds, this scaffold enhanced wound healing with no scaring and improved tissue quality [102].

#### 4.2.3. Lipid-Based Nanoparticles

Lipid nanoparticles include similar structures such as micelles, reverse micelles, emulsions, microemulsions, transferosomes, ethosomes liposomes, and solid lipid nanoparticles (SLN). These structured systems have been broadly used to release several active compounds, including chemotherapeutic drugs [103], antibiotics [104], and genetic material [105,106]; however, in the last years’ diverse studies have described that SLN present an improved capacity to reach the dermis [107]. Different lipidic systems have been used to deliver a plethora of compound to the dermis; in this work, we describe a few applications in which lipid nanoparticle has been utilized to deliver compound with pharmaceutical potential into the dermis. Nanostructured lipid carriers (NLC) have been applied to deliver capsaicin to the dermis with the intent of reducing the irritation associated with the application of raw formulations [108]. Furthermore, Ghasemiyeh et al. loaded cyproterone acetate (CPA) in nanostructure lipid carriers with different sized (100–600 nm). They demonstrated that encapsulated CPA into lipid carriers presented a better penetration than free CPA. The optimal penetration to epidermis-dermis layers via follicular appendage was found for lipids with a 300 nm size [81].

However, in treating immunologically related pathologies, SLN has been used to encapsulate atorvastatin, generating a system that prevents the systemic absorption of this drug and prolongs the anti-inflammatory effect in the treatment of scalp seborrheic dermatitis [109]. Additionally, metformin’s anti-inflammatory and ROS reduction capacity, a drug commonly prescribed to treat diabetes, has been studied to improve the treatment of skin inflammatory pathologies. SLN load with metformin particles has shown the capacity to reach the deeper section of the dermis, increasing the dermal concentration of metformin [110]. Another anti-inflammatory application of SLN has been reported with cyclosporine A (CsA), the SLN@CsA particles present an improved retention and penetration into the dermis in comparison to the application of a suspension of CsA [111]. The traditional non-steroidal anti-inflammatory drug (NSAIDs) ibuprofen has been encapsulated using SLN. Those SLN@Ibuprofen particles showed an improved performance in treating skin inflammation in a murine model compared with the topical application of gel-based ibuprofen; additionally, this formulation presents a release profile related to the pH showing an increased liberation rate at pH 7.4, which is the pH present in the dermis [105,112,113]. Other applications of lipid-based nanoparticles oriented toward the cosmetic field have been explored [114], where SLN loaded with vitamin A (Vit-A) has been studied for dermal application, showing that SLN presents a higher load capacity of Vit-A and an increased penetration of the particle and distribution of Vit-A compared to a Vit-A suspension gel [115].

## 5. Metal-Organic Frameworks as an Integrative Tool for Skin Treatments 

**Figure 3:**
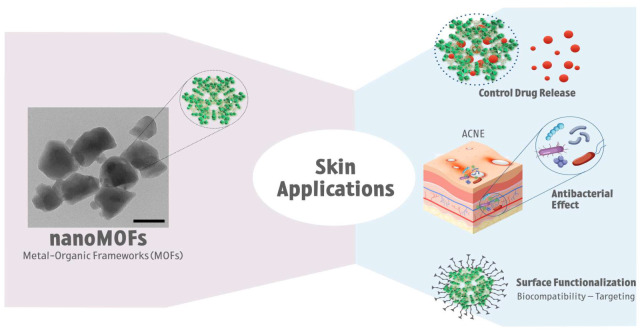
Morphological structure of MIL100 metal-organic frameworks (MOFs) acquired by electronic transmission microscopy (TEM), and some schematic representations of MOFs applications on the skin.

Metal–organic frameworks (MOFs) are well-defined three-dimensional porous solids assembled from inorganic metal nodes connected by multitopic organic ligands. They present structural flexibility, large surface areas, and pore sizes that can be tailored by a combination of metals, ligands, and synthesis conditions for a given application [116]. Numerous applications in many fields are being developed, such as gas storage [117], separation [118], chemical sensing [119], catalysis [120], and potential biomedical applications, including drug storage and delivery [121,122], biomedical gas storage [123], biosensing [124,125], or molecular imaging [126]. Figure 3 describes a morphological structure of MIL100 acquired by transmission electronic microscopy and illustrates some skin applications of metal–organic frameworks (MOFs).

The first obstacle to using MOFs in skin treatments based on dermis therapeutic targets is penetrating across the epidermal layer by some of the routes mentioned above. As a combination of inorganic and organic materials, MOFs allow the incorporation as primary building blocks of cations (Au^+^, Ag^+^, Cu^2+^, or Zn^2+^) involved in diverse biological processes and bioactive ligands as organic connectors. Thus, the progressive degradation of the MOF framework can be an effective therapy for human pathogenic bacteria causing various infections and syndromes in the skin. A completely bioactive MOF constructed with Zn^2+^ and azelaic acid (Az) coordinated to the metallic centers (BioMIL-5) presented interesting antibacterial and dermatological properties for treating several skin disorders [127]. Recently, BioMOFs based on an alkaline element (K^+^) and Az showed superior antibacterial activity against *Staphylococcus epidermidis* and *Staphylococcus aureus* than azelaic acid [128]. Antibiotic Carbenicillin (Car) was coordinated with Ga^3+^ to form a pH-sensitive MOF and used to coat hollow TiO_2_ nanoshells. Under an acidic microenvironment at infected sites, the MOF gradually degrades, releasing Car and Ga^3+^ in combination with the ROS (Reactive Oxygen Species) generator TiO_2_ exhibiting an effective simultaneous inhibition of *Pseudomonas aeruginosa* (PA) and methicillin-resistant *Staphylococcus aureus* (MRSA), supporting that strategy as a potential antibacterial alternative to fight against these relevant pathogens [129]. A pH-responsive core-shell nano assembly has been developed using core mesoporous silica nanoparticles (MSN) loaded with *β*-lactamase inhibitor (sulbactam) coated with a pH-responsive MOF based on the antibiotic Car and Fe^3+^. This nano assembly was stable under physiological conditions; however, at lower pH, the MOF on the MSN surfaces degrades gradually, releasing their components and unblocking the MSN pores, which led to the release of the trapped inhibitor [130]. However, an excess of metal ions released may also be dangerous as it can negatively affect the biological processes where they are involved, in addition to bacteria. The in-situ incorporation of folate inside the pores of the Cu-based MOFs HKUST-1 framework increases the hydrophobicity of HKUST-1 pores, which prevents proteins and water molecules diffuse to the Cu^2+^ sites from hampering the break of Cu-carboxylate linkages [131]. Additionally, the presence of the vitamin reduced the cytotoxicity towards human dermal fibroblasts. 

An alternative route to creating effective antibacterial agents based on MOFs is the possibility of attaching additional organic fragments by covalent post-synthetic modifications in the organic ligands. A series of MOF/Ce-based nanozymes have been developed using a peroxidase-like activity of Au-doped MIL-88B(Fe) MOFs. The ligands located at the external surfaces were modified with Ce-NTA ((1S)-N-(5-amino-1-carboxypentyl)iminodiacetic acid) complexes that presented DNase-mimetic activity to catalyze the hydrolysis of extracellular DNA. In vivo tests revealed that cumulative effects of dual enzyme-like MOFs on treated subcutaneous abscesses demonstrated a significant reduction of inflammatory cells and improved wound healing [132]. On the other hand, MIL-88B(Fe)-based nanozymes presented a relatively lower catalytic activity. A strategy to increase their therapeutic activity was modifying the MOF’s external surfaces with a COF (Covalent Organic Framework), creating a MOF @COF hybrid. The COF proportionated an external morphology proper to catch bacteria and presented a microenvironment close to the MOF catalytic sites capable of activating the substrates via non-covalent interactions [133]. 

The presence of unsaturated metal nodes at the external MOFs surfaces provides opportunities for their surface engineering. This surface modification of MOFs improves their colloidal stability, permits the control of the release of the ions, guests, or ligands, and tunes the hydrophobic/hydrophilic character of their external surfaces, improving their applicability. It can form composites in which MOF particles are embedded into functional (co)polymers with intrinsic bactericidal properties that protect MOFs from a fast degradation in physiological fluids. Additionally, as a strategy, the inherent properties of those polymers can be exploited. Studies based on films as crosslinking agents from HKUST-1 and CS exhibited a relatively slow release of copper ions, efficient antibacterial activity to *Staphylococcus aureus*, and negligible biotoxicity [134]. The incorporation of HKUST-1 within citrate-based hydrogels enables a sustainable copper ions release to maintain antioxidant and thermoresponsive hydrogel properties [135]. Studies of ZIF8@PVA (polyvinylalcohol) hydrogel omniphobic membranes also allow the controllabe release of Zinc ions [136]. 

Efficient wound dressing can be created by the sustained release of non-toxic amounts of calcium, copper, and zinc ions from a combination of Zn^2+^ and Cu^2+^ ions in MOFs-niacin and MOFs encapsulated in alginate microcapsules [137]. The photothermal properties of MOFs (Prussian Blue) embedded into CS-based hydrogels can be exploited as an antibacterial agent through the synergistic effect of heat and the electropositive surface from hydrogel [138]. Ag-based MOF composites with CS can act as barriers to the permeation of bacteria to wounds due to their antibacterial activities and can also release trace amounts of Ag^+^ to reduce inflammation and accelerate wound healing [139]. In addition to the release of their primary components due to their progressive degradation MOFs provide sites to be loaded with biologically active substances such as gases, organic molecules, ions, enzymes, or nanoparticles [140].

Nitric oxide (NO) is an essential molecule with well-recognized therapeutic properties [141]. Pinto et al., developed novel vitamin B3 MOFs with Ni and Co as metal centers and titanium carboxylate MIP-177 for NO storage and demonstrated possible therapeutic applications of the NO release [142,143]. The liporeductor cosmetic caffeine (Caf) was loaded into MIL100, and nanoparticles were embedded into biocompatible polymers (PVA) and gelatin. Caf was progressively released from the composite and could permeate through the skin, reaching the targeted adipocyte region, paving the way for the topical administration of MOF polymer-based devices for the cutaneous or transdermal administration [144]. 

Photosensitive properties of MOFs also can be exploited. Zirconium-based PCN-224 MOFs loaded with Ag^+^ ions and coated with HA were tested with MRSA bacteria. The combination of ROS from PCN-224 organic ligands and the release of Ag ions-Ag showed a much higher antibacterial activity effect than separated PCN-224Ag and silver ions. MFM-300(Sc) MOFs can be excellent drug carriers for the transdermal administration of natural antioxidant ferulic acid (FA), which has a protective role for the main skin structures such as collagen, fibroblasts, keratinocytes, and elastin [145]. Recently, Taherzade et al. [146], created topical patches based on water-stable and biosafe Fe carboxylate MOFs (MIL-100 and MIL-127), the biopolymer polyvinyl alcohol (PVA), and the selective adsorption of two co-encapsulated drugs used in skin disorders (azelaic acid (Az) as antibiotic, and nicotinamide (Nic) as anti-inflammatory), to develop an advanced cutaneous combined therapy. MOFs for cutaneous applications can be further optimized for combined treatments using both progressive framework degradation and slow delivery of active substances. A dual cooperative controllable release system has been designed by incorporating small molecular drugs (dimethyloxalylglycine, DMOG) into ZIF-67 nanoparticles. The strategy used to perform a controlled release of DMOG and Co^2+^ ions included the nanoparticles in a micro-patterned poly (L-lactic acid) PLLA/Gellatin nanofibrous scaffolds. Synergistic effects that promoted accelerated healing in diabetic chronic wounds were found [147]. HKUST-1 MOFs have been utilized in the design of multifunctional antimicrobial agents through the delivery of antibacterial gases, antibiotic molecules, and antibacterial metal ions at different rates as a strategy that permits both fast and long-lived bactericidal action [148]. 

## 6. Conclusions and Future Perspectives

The progression of pathologies and symptoms, such as inflammation, allergies, psoriasis, and dermatitis, are directly related to alteration in the dermis’ cellular communication and maintenance of matrix components. However, most literature based on nanoparticle formulations to treat skin conditions is commonly targeted to trespass de SC and reach the epidermis, the external layer of the skin. Our revision aims to illustrate how the integrity of the dermis is essential to maintain the skin’s protective functions and to attain a significant improvement in treating pathologies. The diverse works summarized in this article prove that targeting the dermis is essential to produce a promising treatment based on nanotechnology. Indeed, the main challenge for dermis drug delivery is the stratum corneum as a physical barrier. Several types of nanoparticles (inorganic, polymeric, or lipidic) have been developed in recent years to circumvent this issue. Those nanoparticles are selected depending on the route chosen to cross the epidermis or considering the chemical properties of the cargo molecule being studied. However, the nanoparticle composition must be considered in the rational strategy to reach an adequate therapeutical effect in the targeted region. Some components, such as metals, can control microbial growth on damaged skin or reduce ROS production. Likewise, some polymers can stimulate the fibroblast to produce extracellular matrix components, contributing considerably to improving the dermis function.

In this context, MOF appears with unique characteristic of inorganic/organic composition that integrate the properties of metals and organic compounds in a unique system; MOFs also have a clear structure, adjustable pore size and shape, excellent surface area and porosity, easy chemical functionalization and can be prepared in large amounts. Despite the numerous articles published on (nano)MOFs, the development of the new (nano)MOFs centered on the dermis as a therapeutic target has not reached their maturity because the full potential of (nano)MOFs has not been exploited yet. MOFs present some drawbacks: They present some degree of matrix degradation, which induces premature drug leakage before reaching the target; their syntheses are often rigorous with high pressures and/or temperatures or involve toxic solvents. Another obstacle is the intrinsic toxicity of the MOF primary components. All these issues reduce their applicability in biomedical applications. Thus, the near future challenge passes from creating MOFs-based nanoparticles whose characteristics permit precise control of the target and drug release without loss of sturdiness. Additionally, it is necessary to carefully investigate the toxicity of the primary building blocks and metals and the biocompatibility issues. However, the most ambitious future approach is the creation of totally bioactive (nano)MOFs in which both metal and organics have an active role.

## Figures and Tables

**Table 1:** Applications of different types of nanoparticles for pathophysiological skin conditions based on dermis strategies.

Type of Nanoparticle	Formulation	Aims	Reference
Inorganic	SiO/peptide	Reduce the inflammatory response liberating the natural compound from the nanoparticle mesoporous.	[73]
Inorganic	ZnO	Prevent microbial infection and decrease the ROS level on the skin.	[74]
Inorganic	Nanocomposite of zinc and silver nanocomposite	Promotes the healing of skin wounds	[75]
Inorganic	Super paramagnetic Iron Oxide/polyethelyimine	Skin penetration via follicular pathways	[76]
Polymeric	Polycaprolactone/gum arabic/ZnO nanocomposite	Promote the regeneration of skin tissue and treat difficult healing skin wounds.	[77]
Polymeric	Dexamethasone loaded dentritic nanoparticle	Reduce the inflammatory response of atopic dermatitis.	[78]
Polymeric	SIlibilin encapsulated in gellan gum	Platform for skin delivery.	[79]
Lipidic	Raloxifene-loaded cubosomes	Transdermal delivery	[80]
Lipidic	Cyproterone encapsulated into nanostructure lipid carriers	Promote penetration via follicular appendages.	[81]
